# A monoclonal antibody against the Wnt signaling inhibitor dickkopf-1 inhibits osteosarcoma metastasis in a preclinical model

**DOI:** 10.18632/oncotarget.8522

**Published:** 2016-03-31

**Authors:** Seth D. Goldstein, Matteo Trucco, Wendy Bautista Guzman, Masanori Hayashi, David M. Loeb

**Affiliations:** ^1^ Division of Pediatric Oncology, Sidney Kimmel Comprehensive Cancer Center, Johns Hopkins University, Baltimore, MD, USA; ^2^ Department of Pediatrics, Section of Hematology-Oncology, Baylor College of Medicine, Houston, TX, USA

**Keywords:** sarcoma, metastasis, Wnt signaling, DKK-1, mouse model

## Abstract

The outcome of patients with metastatic osteosarcoma has not improved since the introduction of chemotherapy in the 1970s. Development of therapies targeting the metastatic cascade is a tremendous unmet medical need. The Wnt signaling pathway has been the focus of intense investigation in osteosarcoma because of its role in normal bone development. Although the role of Wnt signaling in the pathogenesis of osteosarcoma is controversial, there are several reports of dickkopf-1 (DKK-1), a Wnt signaling antagonist, possibly playing a pro-tumorigenic role. In this work we investigated the effect of anti-DKK-1 antibodies on the growth and metastasis of patient-derived osteosarcoma xenografts. We were able to detect human DKK-1 in the blood of tumor-bearing mice and found a correlation between DKK-1 level and tumor proliferation. Treatment with the anti-DKK-1 antibody, BHQ880, slowed the growth of orthotopically implanted patient-derived osteosarcoma xenografts and inhibited metastasis. This effect was correlated with increased nuclear beta-catenin staining and increased expression of the bone differentiation marker osteopontin. These findings suggest that Wnt signaling is anti-tumorigenic in osteosarcoma, and support the targeting of DKK-1 as an anti-metastatic strategy for patients with osteosarcoma.

## INTRODUCTION

Osteosarcoma is the most common bone tumor of adolescents and young adults [1, 2]. Surgery alone cures only a small minority of patients who present with localized disease [3, 4]. The introduction of systemic chemotherapy resulted in rates of long term survival approaching 75% in patients with localized osteosarcoma, but has had a minimal impact on the survival of patients who present with metastatic disease [5, 6]. Numerous clinical trials with increasingly intensive chemotherapy regimens have failed to improve survival rates for this population, which has led to a focus on understanding the biology of osteosarcoma metastasis in the hopes that this will lead to the development of new approaches targeting metastasis-specific cellular pathways [4].

The Wnt signaling pathway has been the focus of intense investigation in osteosarcoma because of its role in normal bone development. The Wnt family is composed of 19 secreted glycoproteins that are required for, among other things, skeletal development and homeostasis [7]. Wnt ligands bind to transmembrane receptors, including the 10 members of the Frizzled family of G protein coupled receptors (which mediate β-catenin-dependent, or canonical signaling) and the receptor tyrosine kinases ROR1 and ROR2 and the receptor tyrosine kinase-like receptor RYK (which mediate so-called noncanonical signaling) [8]. The extraordinary complexity of the Wnt signaling system is further complicated by the existence of secreted Wnt antagonists such as the secreted frizzled-related proteins and Wnt inhibitory factor 1 (WIF1), which bind Wnt proteins, and sclerostin and the dickkopf family of proteins, which interact with Wnt receptors [7]. Canonical, β-catenin-dependent Wnt signaling enhances osteoblastogenesis and bone formation and decreases osteoclastogenesis and bone resportion [7]. Interestingly, activation of noncanonical signaling by Wnt5a binding to ROR2 enhances osteoclastogenesis and bone resorption [9], while Wnt5a signaling through the G-protein-linked activation of Protein Kinase Cδ induces osteoblastogenic differentiation of murine mesenchymal stem cells [10].

The role of Wnt signaling in the pathogenesis of osteosarcoma is unclear. Although Wnt signaling has been implicated as a driver of osteoblast differentiation, other work has suggested that Wnt signaling may also drive proliferation of osteosarcoma cells. For example, Kansara and colleagues reported that WIF1 is epigenetically silenced in human osteosarcoma cell lines [11]. *In vitro*, WIF1 suppresses β-catenin expression in osteosarcoma cell lines and induces differentiation of primary human osteoblasts, and in primary human osteosarcoma samples, silencing of WIF1 is associated with increased proliferation, increased β-catenin expression, and loss of differentiation, implying that de-repression of Wnt signaling plays a positive role in osteosarcoma pathogenesis. In similar work, Zhao et al performed microarray analysis of an osteosarcoma genetically engineered mouse model, comparing localized *vs* metastatic tumors and found downregulation of NKD2, a negative regulator of Wnt signaling, in metastatic tumors compared with localized tumors [12]. Overexpression of NKD2 in osteosarcoma cell line decreased proliferation, migration, and invasion *in vitro* and diminished tumor growth and metastasis *in vivo*, consistent with a model wherein Wnt signaling potentiates these aggressive behaviors in osteosarcoma. In striking contrast, there are several reports of dickkopf-1 (DKK-1), also a Wnt signaling antagonist, possibly playing a pro-tumorigenic role in osteosarcoma.

The dickkopf family of proteins are secreted glycoproteins that have an allosteric inhibitory effect on LRP 5/6, which the Wnt/Frizzled complex requires to inactivate axin and release β-catenin in the canonical Wnt pathway [13]. The group comprises five molecules; DKK-1 through DKK-4 and a DKK-3-related protein called Soggy [14]. The best studied of this group is DKK-1. The hallmark of DKK-1, -2, and -4 is their inhibition of Wnt signaling, though DKK-2 may activate Wnt signaling in select circumstances [15]. These DKKs play a role in vertebrate development, particularly axial patterning- Dickkopf is German for “big head” [16]. DKK-3 and Soggy appear to be evolutionarily divergent and do not play a role in Wnt signaling [15].

In the adult, DKK-1 is implicated in bone formation and bone disease, particularly multiple myeloma and Pagets disease of bone. Additionally, there have been investigations into its role in bone cancers. Gregory et al reported that as human mesenchymal stem cells begin to proliferate rapidly in culture, they secrete high levels of DKK-1, and that antibodies against DKK-1 slow proliferation [17]. This same group noted elevated serum DKK-1 levels in osteosarcoma patients, and using immunohistochemistry demonstrated high levels of DKK-1 expression in human osteosarcoma samples, concentrated at the proliferative, invading edge of tumors [18]. They also reported that DKK-1 reduced osteogenic differentiation of human mesenchymal stem cells, and that immunodepletion of DKK-1 attenuated the effect. More recently, Krause and colleagues reported that MOS-J osteosarcoma cells engineered to constitutively overexpress DKK-1 are impaired in their ability to differentiate, both *in vitro* and *in vivo*, and that these cells are more proliferative and form larger, more destructive tumors upon orthotopic implantation [19].

In light of the reports that DKK-1 can be measured in the serum of osteosarcoma patients, that DKK-1 can inhibit osteoblastic differentiation, and that overexpression of DKK-1 appears to induce a more aggressive phenotype in osteosarcoma cells implanted in an orthotopic location, we investigated DKK-1 expression in an orthotopic patient-derived xenograft model of osteosarcoma as well as the effect of anti-DKK-1 antibodies on growth and metastasis of patient-derived osteosarcoma xenografts. We were able to detect human DKK-1 in the blood of tumor-bearing mice and found a correlation between DKK-1 level and tumor proliferation. Treatment with the anti-DKK-1 antibody, BHQ880, slowed the growth of orthotopically implanted patient-derived osteosarcoma xenografts and inhibited metastasis. This effect was correlated with increased nuclear beta-catenin staining and increased expression of the bone differentiation marker osteopontin.

## RESULTS

### Correlation between serum DKK-1 levels and osteosarcoma growth

**Figure 1:**
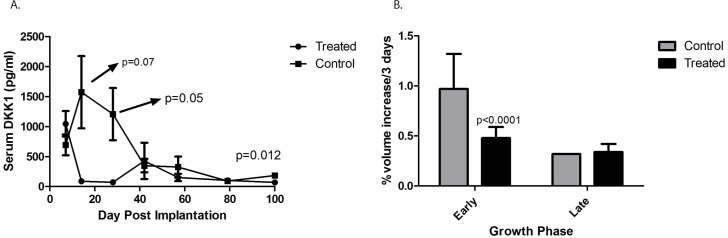
Serum levels of DKK-1 in an osteosarcoma xenograft **A.** Significant levels of human DKK-1 (ranging from 500 to 2200 pg/ml) were detected in the serum of tumor-bearing mice over the first 4 weeks, but surprisingly levels fell to nearly undetectable as tumors grew larger. **B.** When the growth rate of the tumors was analyzed prior to 50 days (“Early”) during the time when serum DKK-1 levels were high tumors were found to grow at a much more rapid rate (averaging almost 1% increase in volume every 3 days). This slowed during the “Late” phase, (to 0.35% volume increase every 3 days), when serum DKK-1 levels were low (*p* < 0.0001). “Treated” refers to those mice administered BHQ880, and “Control” refers to those mice who were not.

Lee et al have previously reported that serum DKK-1 levels are elevated in children with osteosarcoma. (18) We measured levels of human DKK-1 in the serum of mice implanted with human osteosarcoma patient-derived xenografts. Fragments of osteosarcoma PDX designated DAR were implanted subcutaneously in the flanks of NSG mice, and blood was drawn every 2 weeks. Serum DKK-1 levels were measured using a human-specific ELISA assay. Significant levels of human DKK-1 (ranging from 500 to 2200 pg/ml) were detected in the serum of tumor-bearing mice over the first 4 weeks, but surprisingly levels fell to nearly undetectable as tumors grew larger (Figure 1A). We evaluated the growth rate of these tumors during the time when serum DKK-1 levels were high and during the time when levels were low. Tumors grew at a much more rapid rate during the first 6 weeks (averaging almost 1% increase in volume every 3 days), while DKK-1 levels were high, and slowed thereafter (to 0.35% volume increase every 3 days), when levels were low (*p* < 0.0001; Figure 1B).

### BHQ880 slows the growth of osteosarcoma xenografts

To determine whether there is a causal relationship between DKK-1 levels and tumor growth velocity, a cohort of mice with subcutaneous osteosarcoma xenografts was treated in parallel with BHQ880, a human neutralizing IgG1 anti-DKK-1 monoclonal antibody [20]. In mice treated with BHQ880, serum DKK-1 levels rapidly fell to undetectable levels that were statistically significantly lower than untreated mice (Figure 1A). Interestingly, there was a substantial, statistically significant decrease in tumor growth velocity in the BHQ880-treated mice (0.47% volume increase every 3 days, compared with 0.95% in control mice, *p* < 0.0001; Figure 1B), but only during the early phase of tumor growth, when control mice had elevated serum DKK-1 levels. During the late phase, when control animals had undetectable serum DKK-1, tumor growth velocity was unaffected by treatment with BHQ880.

**Figure 2:**
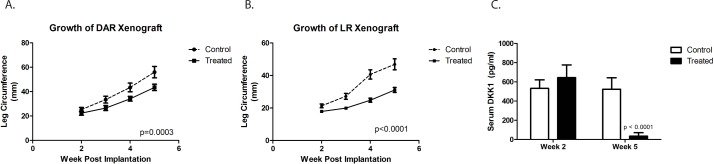
DKK-1 antibody BHQ880 slowed the growth of two different orthotopically implanted osteosarcoma xenograft, DAR (**A.**) and LR (**B.**). Using an ELISA assay on serum at two different time points, we confirmed that BHQ880 also rendered serum DKK-1 levels undetectable in treated mice (**C.**).

Our previous work demonstrated that growth and metastatic propensity of our osteosarcoma PDXs is influenced by site of implantation [21]. We therefore investigated whether site of implantation could affect our ability to detect DKK-1 in the serum of tumor-bearing mice and whether BHQ880 affects the growth of osteosarcoma PDX implanted in an orthotopic location. One cohort of mice had the DAR PDX implanted in the pretibial space, and a second had the LR PDX implanted in the pretibial space. After 2 weeks, half of the mice in each cohort began treatment with BHQ880. At the time of treatment initiation, all mice had detectable levels of human DKK-1 in their serum. As we saw with subcutaneous xenografts, BHQ880 slowed the growth of orthotopically implanted DAR and LR xenografts (Figure 2A and 2B). Using the same ELISA assay, we confirmed that BHQ880 also rendered serum DKK-1 levels undetectable in treated mice (Figure 2C).

### BHQ880 decreases osteosarcoma metastases

**Figure 3:**
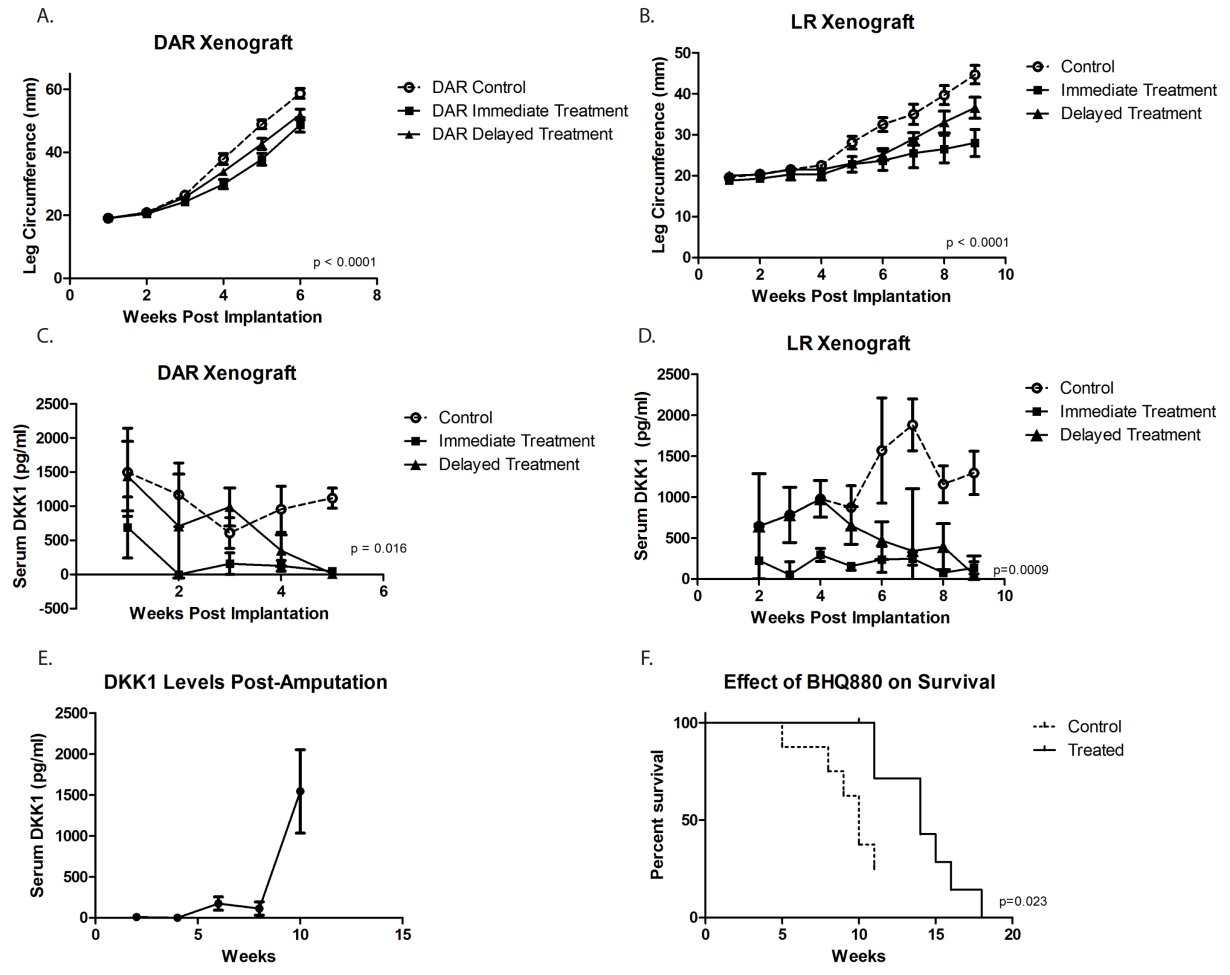
Using an orthotopic implantation/amputation model for DAR (**A.**) and LR (**B.**), treatment with BHQ880 slowed the growth of both tumors, and this growth inhibition was seen whether treatment was initiated immediately or only after the development of a palpable tumor (Delayed Treatment). Serum DKK-1 levels diminished to undetectable levels in all treated mice, whether treated immediately or upon development of palpable tumor (**C.** and **D.**). Hindlimbs were resected from mice when leg diameter reached 20 mm, and mice were subsequently observed for the development of metastases. Among the mice that were ultimately found to have metastatic disease, a sharp increase in serum DKK-1 levels was noted just prior to the development of clinical signs of metastasis or death. (**E.**). In the LR model (unable to assess in DAR due to administrative censoring), treatment with BHQ880 resulted in prolonged survival (**F.**).

**Figure 4:**
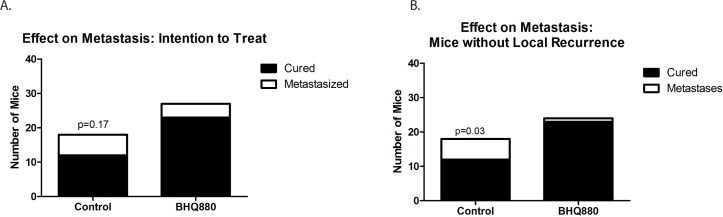
Six of the 18 untreated mice, but only 4 of the 27 mice treated with BHQ880 developed distant metastases **A.** This difference approaches, but does not reach, statistical significance (*p* = 0.17 by Fisher's Exact Test). Excluding 3 mice with local recurrence, only one of the 24 BHQ880-treated mice developed metastatic disease, compared with 6 of 18 control mice **B.**, which is a statistically significant difference (*p* = 0.03, Fisher's Exact Test).

We next utilized our orthotopic implantation/amputation model of osteosarcoma metastasis (20) to determine whether, in addition to slowing tumor growth, BHQ880 might also inhibit metastasis. NSG mice had either the DAR or the LR PDX implanted in the pretibial space. One week after implantation, mice were randomly divided into 3 groups: control, immediate treatment, or delayed treatment (treatment initiated when a tumor was palpable). Serum DKK-1 levels were monitored weekly and leg circumference was measured weekly to monitor tumor growth. As seen in the prior experiment, treatment with BHQ880 slowed the growth of both PDX models, and this growth inhibition was seen whether treatment was initiated immediately or only after the development of a palpable tumor (Figure 3A and 3B). Serum DKK-1 levels diminished to undetectable levels in all treated mice, whether treated immediately or upon development of palpable tumor (Figure 3C and 3D). Hindlimbs were resected from mice when leg diameter reached 20 mm, and mice were subsequently observed for the development of metastases. Among the mice that were ultimately found to have metastatic disease, a sharp increase in serum DKK-1 levels was noted just prior to the development of clinical signs of metastasis or death. (Figure 3E). To evaluate a potential effect on the establishment of subclinical metastatic disease, mice implanted with the DAR model were sacrificed 10 weeks postoperatively and subjected to necropsy. To evaluate a potential effect on disease-related survival, mice implanted with the LR model were not euthanized until they were noted to be in acute distress or have a body condition score of 1. In the LR PDX model, treatment with BHQ880 resulted in prolonged survival (Figure 3F). Evaluating all of the mice in this experiment revealed that among those treated immediately after tumor implantation, no metastases were seen and only a single mouse, implanted with the DAR PDX, developed a local recurrence. Only 1 of the 5 mice implanted with the LR PDX and treated in delayed fashion (after growth of palpable tumor), and none of the 5 treated immediately, developed pulmonary metastases, compared with 3 of the 10 control mice. Two of the control mice also developed intraabdominal metastases. Three of the 12 mice implanted with the DAR PDX (all in the delayed treatment group) suffered local recurrences, and these were the only 3 mice to develop metastases (both lymphatic, to ipsilateral axillary nodes, and hematogenous to the kidney, ovary, and peritoneum). Thus, discounting the mice with local recurrence (presumably reflecting a failure of the surgery to remove the entire primary tumor, which clearly increases risk of metastasis compared with mice whose resections removed the entire tumor burden), only one of the 24 BHQ880-treated mice developed metastatic disease, compared with 6 of 18 control mice, which is a statistically significant difference (Figure 4, Table 1, *p* = 0.03, Fisher's Exact Test).

**Table 1:** Post-amputation outcomes of mice treated with or without BHQ880

PDX Model	Treatment	N	Local Recurrence	Any Distant Spread	Lymphatic Metastasis	Hematogenous Metastasis
LR	Control	10	0	4 (40%)	0	4 (40%)
	Immediate	5	0	0	0	0
	Delayed	5	0	1 (20%)	0	1 (20%)
DAR	Control	8	0	2 (25%)	0	2 (25%)
	Immediate	5	1 (20%)	0	0	0
	Delayed	12	3 (25%)	3 (25%)	3 (25%)	3 (25%)

NSG mice were implanted with either the LR or the DAR PDX. Mice were then treated either immediately with BHQ880, or in a delayed fashion, after development of a palpable tumor. Control mice were not treated. In the DAR-implanted, delayed treatment cohort, the 3 mice which developed local recurrences all also developed both lymphatic and hematogenous metastases.

### Treatment with BHQ880 increases Wnt pathway activity in an osteosarcoma xenograft, increasing differentiation

**Figure 5:**
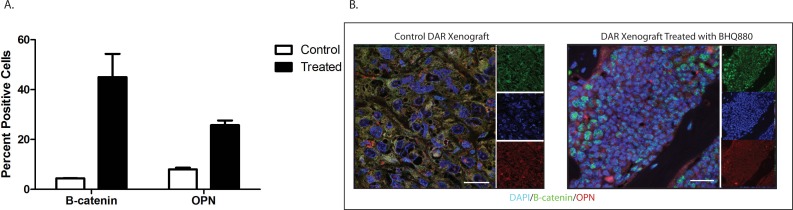
Immunofluorescence studies of the DAR xenograft excised from control and BHQ880-treated mice In control mice, only 4.4% of tumor cells had nuclear β-catenin staining, compared with 45% of cells in tumors from treated mice. Osteopontin staining was detected in only 8% of tumor cells from control mice, compared with 25.8% of tumor cells in treated mice.

**Figure 6:**
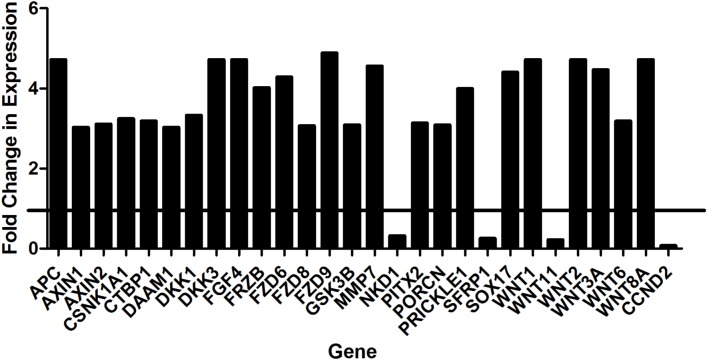
Significant increases in the levels of expression of multiple Wnt-related genes were detected in BHQ880-treated tumors compared with control

To clarify the mechanism by which BHQ880 inhibits osteosarcoma metastasis, we investigated the DAR osteosarcoma PDX in more detail. Because DKK-1 is thought to act by inhibiting β-catenin-dependent (canonical) Wnt signaling, BHQ880 would be expected to increase nuclear localization of β-catenin. We therefore performed immunofluorescence studies of the DAR xenograft excised from control and BHQ880-treated mice. In control mice, only 4.4% of tumor cells had nuclear β-catenin staining, compared with 45% of cells in tumors from treated mice (Figure 5). To confirm that the increase in nuclear β-catenin has functional significance, we investigated the expression of a panel of Wnt-associated genes in tumors grown in control and treated mice. NSG mice had the DAR xenograft implanted in an orthotopic location. When the extremity tumor reached palpable size, the mice were randomized to either BHQ880 or vehicle control for 2 weeks (6 doses). Tumors were harvested, and the expression of a panel of Wnt target genes was evaluated using a Wnt signaling PCR array. As expected, we found significant increases in the levels of expression of multiple Wnt-related genes in BHQ880-treated tumors compared with control, and decreases in the expression levels of only 4 genes (Figure 6). Finally, to test our hypothesis that increased Wnt signaling inhibits metastasis by increasing the differentiation status of osteosarcomas, we evaluated expression of osteopontin, a marker of bone differentiation, in DAR tumors grown in control and in treated mice. Interestingly, we detected osteopontin staining in only 8% of tumor cells from control mice, compared with 25.8% of tumor cells in treated mice (Figure 5). Thus, treatment with BHQ880 leads to increased nuclear β-catenin localization which drives increased Wnt signaling, leading to increased differentiation and decreased growth and metastasis.

**Figure 7:**
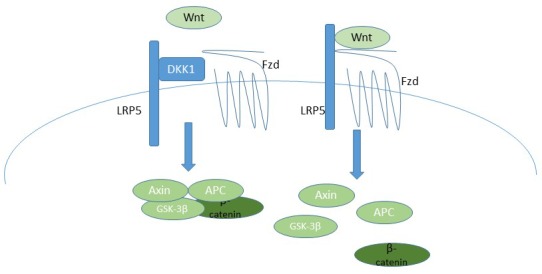
A schematic of the canonical Wnt signaling pathway, indicating how DKK-1 inhibits pathway activation, and thus how BHQ880 would activate signaling On the left, DKK-1 blocks the formation of the LRP5/Fzd heterodimer, resulting in maintenance of the Axin/APC/GSK-3β/β-catenin complex, which mediates proteosomal degradation of β-catenin. In the absence of DKK-1, on the right, Wnt binds the LRP5/Fzd receptor, resulting in dissolution of the Axin/APC/GSK-3β/β-catenin complex, which allows β-catenin to translocate into the nucleus and initiate transcription of target genes.

## DISCUSSION

Although the introduction of systemic chemotherapy dramatically improved the survival of patients with localized osteosarcoma, metastatic recurrence remains a significant problem. Between 25 and 40% of patients with localized disease will suffer a metastatic recurrence, and up to 75% of these patients will die of their disease [22-25]. These numbers have not significantly changed since the 1980s, despite numerous clinical trials aimed at improving the efficacy of chemotherapy. Thus, a biologically based therapy that can inhibit osteosarcoma metastasis is urgently needed. Our work suggests that BHQ880, a monoclonal antibody against the Wnt signaling inhibitor, DKK-1, might be such a drug, and that circulating DKK-1 may be a biomarker of osteosarcoma. We found that human DKK-1 can be detected in the blood of mice implanted with human osteosarcoma patient-derived xenografts, that treatment with BHQ880 dramatically reduces serum DKK-1 levels, modestly decreases primary tumor growth, and dramatically inhibits the development of metastases in mice whose tumors are completely resected.

The role of Wnt signaling in sarcoma biology remains controversial, with some studies suggesting an oncogenic role for this pathway, and other studies supporting an anti-tumorigenic role [26, 27]. Our work directly addresses this controversy. We found that treatment with BHQ880 causes increased nuclear localization of β-catenin, which results in increased expression of a number of Wnt target genes and leads to increased expression of osteopontin, a marker of bone differentiation (Figure 6). These findings support the idea that the Wnt signaling pathway promotes bone differentiation of osteosarcoma, inhibiting both tumor growth and metastasis. This may also be complementary to prior discoveries that increased DKK-1 production- and thus an inhibition of Wnt- allows proliferation of mesenchymal stem cells and is noted in Paget's disease of bone [28].

Our work with BHQ880 is highly translationally relevant. Traditional preclinical mouse models have not proven adequate to study the biology and treatment of spontaneous distant sarcoma metastasis [29-31]. Our use of a previously validated orthotopic implantation/amputation model, which we believe better recapitulates tumor microenvironment compared to traditional approaches, is a strength of this work. Unlike genetic approaches used to identify potential targets that might inhibit metastasis, our experiments closely mimic the human disease - tumor grows in the leg, treatment begins when a tumor is palpable, tumors are resected, and animals are followed post-operatively for metastases. In this context, treatment with BHQ880 results in a significant decrease in the development of metastases which corresponds with an improved survival. Our work supports further clinical investigation with agents that antagonize DKK-1 signaling and holds out hope that drugs can be developed to specifically target metastasis, finally addressing the major clinical event that limits our ability to cure every patient with osteosarcoma.

A limitation of this study is the reliance on immunodeficient mice, which limits our ability to fully evaluate the role of the immune system in our understanding of metastasis. The contemporary understanding of the “metastatic cascade” is that the following must occur for cancer to spread: invasion into and migration through surrounding tissue, intravasation, anoikis resistance, immune system evasion, extravasation, and proliferation [4, 32]. Although NSG mice do contain myeloid cells, they do not contain lymphocytes or NK cells, which may affect metastatic patterns.

In conclusion, in this work we report the effect of anti-DKK-1 antibodies on the growth and metastasis of patient-derived osteosarcoma xenografts. We were able to detect human DKK-1 in the blood of tumor-bearing mice and we found a correlation between DKK-1 level and tumor proliferation. Treatment with the anti-DKK-1 antibody, BHQ880, slowed the growth of orthotopically implanted patient-derived osteosarcoma xenografts and inhibited metastasis. This effect was correlated with increased nuclear beta-catenin staining and increased expression of the bone differentiation marker osteopontin. These findings suggest that Wnt signaling is anti-tumorigenic in osteosarcoma, and support the targeting of DKK-1 as an anti-metastatic strategy for patients with osteosarcoma.

## MATERIALS AND METHODS

### Establishment of xenograft

All procedures and experiments involving mice were performed according to protocols approved by Johns Hopkins Animal Care and Use Committee. The DAR PDX was created from cells isolated from a malignant pleural effusion in an osteosarcoma patient, and the LR PDX was created from a pulmonary nodule resected from an osteosarcoma patient with a metastatic relapse. Both xenografts were a kind gift from Dr. Chand Khanna (Pediatric Oncology Branch, National Cancer Institute).

For all experiments, 3 mm tumor fragments were implanted into either the subcutaneous flank or into the pretibial space, as previously described. (20) Serially passaged tumor was grown in the hindlimb of a single NOD/SCID/IL-2Rγ-null (NSG) female mouse. When the tumor reached sufficient size, the mouse was sacrificed and the tumor divided into fragments of approximately 3 mm diameter. Intact fragments were washed in Matrigel basement membrane matrix (BD Biosciences, San Jose, CA) and implanted simultaneously into an entire cohort of recently weaned NSG female pups under general anesthesia (ketamine 9 mg/ml and xylazine 1 mg/ml given as 7 cc/kg intraperitoneal injection). When the tumor reached appropriate size (approximately 1.5 cm in maximal diameter), the limb was amputated through the proximal femur and mice were observed post-operatively for recurrence or metastasis.

### Tumor measurements

Tumor size was measured twice weekly beginning when the tumor was clearly palpable, typically at a hindlimb diameter of 7 mm or greater. Limb circumference was estimated using an elliptical formula from hindlimb caliper measurements of *2π√((a^2^ + b^2^)/2)*, where a is the largest leg diameter perpendicular to the bone and b is the diameter in the orthogonal direction. Mice were sacrificed or considered for survival amputation when leg diameter including tumor approached 15 mm in the greatest dimension.

### Quantitative polymerase chain reaction

For RNA analysis, freshly harvested tumor was frozen at −80°C in RNAlater stabilization reagent (QIAGEN Inc, Valencia, CA). RNA was extracted from tissue using the RNeasy Mini Kit according to the manufacturer's instructions (QIAGEN Inc) and reverse transcribed (Iscript Reverse Transcriptase Bio-Rad, Hercules, CA). For quantitative PCR, 1 μl cDNA was mixed with 10 μl SYBER Green SuperMix and appropriate primers. Quantitative PCR was performed using a standard two-step amplification/melt protocol.

### Serum DKK-1 measurement

Serum levels of circulating human DKK-1 were measured by enzyme-linked immunosorbent assay (Quantikine ELISA, R&D Systems, Minneapolis, MN). Mouse blood was collected biweekly by lancet puncture of the facial vein followed by centrifugation and immediate storage of the supernatant at −80°C.

### Anti-DKK-1 antibody

A lyophilized monoclonal antibody to human DKK-1 was obtained from Novartis, Inc (Cambridge, MA). Reconstituted solution was administered *via* intraperitoneal injection at 10 mg/kg three times weekly.

### Immunofluorescence

Formalin-fixed, paraffin-embedded tumor sections were deparaffinized with graded ethanol/xyline, followed by antigen retrieval in sodium citrate pH 6.0 at 60°C for 30 min. After blocking with normal goat serum, sections were incubated overnight at 4°C with one of the following primary antibodies: goat anti-DKK-1 (R&D Biosciences, Cat no. 5439 DK), mouse monoclonal anti-osteopontin, (Abcam, Cambridge, MA, Cat no. ab166709), or rabbit anti-beta catenin (Cell Signaling, Danvers, MA, Cat no. 8480). Double and triple labelling was achieved using donkey anti-goat Alexa 649, donkey anti-mouse Alexa 549, and donkey and goat anti-rabbit Alexa 488 (Jackson Immunoresearch, West Grove, PA), by adding these to the slides respectively and incubating for an hour at room temperature after primary incubation. Sections were washed in TBST (Triton x100 0.1% PBS), coverslipped using fluoromount with DAPI and subsequently analyzed with a Zeiss 710 Confocal microscope at 40 X and 60 X magnification. ZEN software was used for analysis and measurements of beta-catenin and osteopontin expression. To quantify protein expression, 10 high power field images (approximately 100 cells per field) taken from different areas of the tumor were counted and normalized to the total number of cells per field for the 60 X images.

### Statistical analysis

Statistical analyses were made using Prism 5.0 software (GraphPad Software, Inc., La Jolla, CA). A *p* value < 0.05 was considered statistically significant.
